# Effects of increased paternal age on sperm quality, reproductive outcome and associated epigenetic risks to offspring

**DOI:** 10.1186/s12958-015-0028-x

**Published:** 2015-04-19

**Authors:** Rakesh Sharma, Ashok Agarwal, Vikram K Rohra, Mourad Assidi, Muhammad Abu-Elmagd, Rola F Turki

**Affiliations:** Center for Reproductive Medicine, Cleveland Clinic, Cleveland, OH USA; Center of Excellence in Genomic Medicine Research, King AbdulAziz University, Jeddah, Saudi Arabia; KACST Technology Innovation Center in Personalized Medicine at King AbdulAziz University, Jeddah, Saudi Arabia; Obstetrics and Gynecology Department, King Abdulaziz University Hospital, Jeddah, Saudi Arabia

**Keywords:** Paternal age, Infertility, Semen parameters, Reproduction, Genetics, Sperm DNA damage, Telomere length, Aneuploidy, Epigenetics, Offspring, Assisted reproductive techniques

## Abstract

Over the last decade, there has been a significant increase in average paternal age when the first child is conceived, either due to increased life expectancy, widespread use of contraception, late marriages and other factors. While the effect of maternal ageing on fertilization and reproduction is well known and several studies have shown that women over 35 years have a higher risk of infertility, pregnancy complications, spontaneous abortion, congenital anomalies, and perinatal complications. The effect of paternal age on semen quality and reproductive function is controversial for several reasons. First, there is no universal definition for advanced paternal ageing. Secondly, the literature is full of studies with conflicting results, especially for the most common parameters tested. Advancing paternal age also has been associated with increased risk of genetic disease. Our exhaustive literature review has demonstrated negative effects on sperm quality and testicular functions with increasing paternal age. Epigenetics changes, DNA mutations along with chromosomal aneuploidies have been associated with increasing paternal age. In addition to increased risk of male infertility, paternal age has also been demonstrated to impact reproductive and fertility outcomes including a decrease in IVF/ICSI success rate and increasing rate of preterm birth. Increasing paternal age has shown to increase the incidence of different types of disorders like autism, schizophrenia, bipolar disorders, and childhood leukemia in the progeny. It is thereby essential to educate the infertile couples on the disturbing links between increased paternal age and rising disorders in their offspring, to better counsel them during their reproductive years.

## Background

Increased life expectancy, advanced age of marriage, various socio-economic factors and an overall change in role of women in society has led couples to start their family at a later age. The increased accessibility to assisted reproductive techniques has increased the chance of older parents with poor pregnancy outcomes to conceive children, hence, increasing the average paternal age at first childbirth. In comparison to 1993, the paternal age of English fathers has increased by 15% in a period of ten years [1]. Increased paternal age affects testicular function [2], reproductive hormones [3], sperm parameters [4,5], sperm DNA integrity [6], telomere length [7], *de novo* mutation rate [8], chromosomal structure [6,9] and epigenetic factors [10].

These changes negatively affect fertility and reproductive outcomes in older couples, contributing to higher incidences of congenital birth defects [11] and fetal deaths [12]. Increasing male age has also been shown to be associated with numerous disorders like achondroplasia [13], autism [14], schizophrenia and bipolar disorders, [14] among many others. In this review article, we will elaborate on the effects of increasing paternal age at the molecular level as well as examine their implications on clinical outcomes. We hope to raise awareness among both clinicians and older couples to the risks associated with delayed fatherhood, which may compromise their parenthood dreams as well as their quality of life.

### Testicular functions and reproductive hormones

Several studies in previous years have shown association between testicular functions and advancing age [2,15-19]. Handelsman et al. reported a negative association between increasing paternal age and reduction in testicular volume for men >80 years [2]. They also reported a reduction in the size of testis [17]. In a study conducted by Mahmoud et al. it was found that compared to the age group 18–40 years, men aged >75 years had 31% smaller mean testicular volume. Decreased testicular volume is attributed to the decrease in number of Sertoli cells [15]. In addition, Johnson et al. reported the thickening of basal membrane of seminiferous tubules with age [16]. Disturbance in blood supply in senile testis were associated with negative changes in terms of hernia-like protrusions, spermiogenesis and thickness of basement membrane [18].

Increased FSH serum levels and decreasing testosterone levels are the most common clinically relevant alterations associated with male ageing [20]. The decreasing testosterone levels in aging men are linked to andropausal symptoms, such as poor libido, fatigue and loss of cognitive function [21]. Both male sexual function and sexual frequency decrease with age [22-24] and the infertility experienced by many older men may in part be related to the decline in sexual activity.

Leydig cells are responsible for testosterone production. The number of Leydig cells tends to reduce with increasing paternal age [19]. Neaves et al. reported that the average total number of Leydig cell nuclei decrease by half in age group of 50–76 years compared to age group of 20–48 years [19]. Reduced number of Leydig cells plays a key role in incidence and pathogenesis of andropause in aging men [25]. The decreased number of Leydig cells also contribute to reduced levels of total testosterone [26] and free testosterone (1.2%) serum levels in paternal group >50 years.

Wu et al. reported that age-affected testicular atrophy is a result of Hypothalamic-Pituitary-Testicular (HPT) Axis alterations that disturb the functions of various reproductive hormones [27]. Advanced paternal age has also been associated with changes in different hormonal levels. Table 1 summarizes the effect of increasing paternal age on reproductive hormones.

**Table 1:** **Effect of advancing paternal age on reproductive hormones**

**Name of the hormone**	**Levels**	**Type of study**	**Reference**
Dehydroepiandrosterone (DHEA)	**↓**	Longitudinal	[28]
Dihydrotestosterone (DHT)	No Change	Longitudinal	[3]
Estrogen	↓	Cross-Sectional	[29]
Follicle-stimulating hormone (FSH)	↑	Longitudinal	[3,19,20,30,31]
Gonadotropin-Releasing Hormone (GnRH)	↓	Animal	[32]
Luteinizing hormone (LH)	↑	Longitudinal	[3]
Sex hormone-binding globulin (SHBG)	↑	Cross-Sectional	[26]
Testosterone	**↓**	Longitudinal	[3,19,21,33]

### Sperm parameters

Semen analysis is an important first step in the laboratory evaluation of the infertile male. It includes the assessment of the ejaculate volume, sperm concentration, motility, and morphology using WHO criteria [34]. Some studies have shown that with increasing paternal age, semen volume, sperm motility, and the percentage of normal morphology tend to decrease [4,35].

With the introduction of the new 2010 WHO guidelines [36], the normal reference range reported at fifth centile has changed for many of the semen parameters. These include: ejaculate volume from ≥2mL to 1.5 mL; sperm concentration (from ≥20 × 10^6^/mL to 15 × 10^6^/mL), Total sperm count from ≥40 × 10^6^ to 39 × 10^6^; percent motility (from ≥50% to 40%); progressive motility from ≥25% (grade a) to 32% (grade a); morphology (percent normal forms) from 14% according to strict criteria to 4%; vitality (% alive) from 30% to 25%; for performing viability test in semen specimens with poor motility. One of the main features of the new guidelines is the inclusion of the reference ranges and the limits which are significantly lower than those reported in the earlier manuals. It also included data from over 1900 men who recently fathered a child within one year of trying to initiate a pregnancy. However there is much controversy regarding the new reference values and the impact in the management of male infertility [37]. The American Urology association recommends that the initial evaluation should include a reproductive history, and two properly performed semen analysis, followed by extended evaluation if semen parameters are abnormal in the initial evaluation [38]. On the other hand the European Association of Urology (EAU) recommends undertaking male examination if the semen analysis is abnormal [39]. The impact of increasing paternal age as reflected in the semen parameters according to the new criteria remains to be seen and interpreted with caution.

Several mechanisms have been proposed to explain how aging in males may cause changes in semen parameters [40]. These changes can be related to seminal vesicle inadequacy which reduces semen volume or changes in prostate, in terms of prostate atrophy such as reduction in water and protein content which might affect sperm motility and ejaculate volume [40]. Kidd et al. also reported that increasing paternal age is correlated with decrease in ejaculate volume, sperm morphology and motility but not with sperm concentration [40]. Comparing two age groups (30y vs. 50y), a significant difference was reported in semen volume (3%-22%), sperm motility (3% - 37%) and morphology (4% - 18%) [40]. In a study conducted by Hossain et al. it was reported that with increasing paternal age, both sperm volume and sperm count decreased [41]. Similarly, in a large prospective study comprising of 3,729 male partners evaluated for semen quality and age-specific changes, a significant decrease was reported in sperm volume and motility with increasing paternal age [42].

Sperm samples from 5081 men aged between 16 and 72 years were examined for effects of male age on semen parameters [43]. Deterioration in sperm quality and quantity after age 35 was reported with declining probability of pregnancy following intercourse with men >34 years old, when women age factor was eliminated [43]. Another recent study investigated the effects of paternal age on DNA fragmentation, semen quality and chromosomal aneuploidies [4]. Spermatozoa from 140 infertile men between 24–76 years of age and 50 fertile men age group (25–65 years) were examined. The findings of the study illustrated that with increased male age, semen volume and vitality decreased while sperm concentration and diploidy increased [4]. However, no significant difference in the motility, morphology and DNA fragmentation was reported with increasing male age [4].

Similarly, in another study the correlation of men’s age with semen quality and seminal levels of epididymal and accessory gland markers were examined. A statistically significant decrease in semen parameters was reported in men aged 35 years and particularly with those over 46 years. This was associated with an increase in the percentage of dead spermatozoa [44]. Semen samples collected from men aged between 30 years to 40 years showed semen parameters to be inversely related to men’s age. Several other retrospective studies have shown a relation between sperm parameters and age and reported lower semen volume, lower progressive motility and percentage of normal morphology in older men compared to younger men [45-47].

### Age threshold

As mentioned earlier, it was reported that the sperm parameters do not change until males reach the age of 34 years [40]. In a study by Kidd et al. [40], the total sperm count was the first parameter to be affected immediately after a person crossed the 34 year threshold. Sperm concentration as well as the percentage of sperm with normal morphology declined at the age of 40. Sperm motility and semen ejaculate volume declined at the age of 43 years and 45 years respectively [43]. Another study conducted in China examined the semen analysis of 20–60 years old men and showed that age was negatively correlated with progressive motility, vitality, and percentage of normal sperm. Rapid progressive motility and percentage of normal sperm morphology began to decline gradually at age 30 years, and progressive motility began to decrease at age 40 years [48] and defective sperm function [49]. The variation in the results of these studies could be due to the differences in the type of study (prospective versus retrospective) [50]. The variation of results in different studies could be related to sexual abstinence time which was different along with many other factors such as type of study, different age groups, sample size and different ethnicities, biological variability and the fact that semen parameters are poor predictors of male fertility potential [51,52]. A compilation of some of the recent studies and their findings related to different sperm parameters is shown in Table 2.

**Table 2:** **Effect of paternal age on different sperm parameters**

**Type of study**	**Age grouping**	**Effects on sperm parameters**				**Reference**
		**Concentration**	**Morphology**	**Motility**	**Ejaculate volume**	
Prospective	24-76	↑	↓	↓	↓	[4]
	22-80	↓	-	↓	↓	[46]
	22-80	-	-	↓	-	[50]
				All CASA parameters of motility except amplitude of lateral head displacement and beat cross frequency		
	30-50	-	↓	↓	↓	[40]
Retrospective	25-55	-	-	↓	↓	[41]
Prospective	Not specific	-	Not measured	↓	↓	[42]

### Genetics of male aging

#### DNA fragmentation

Some of the potential causes of DNA damage in sperm are abnormal protamination or abnormal protamines compaction [53-55]. It is attributed to the presence of histones (15%) that are not converted into protamines and result in altered P1/P2 ratio in infertile men, protamine deficiency [56-60]. Oxidative stress as a result of increased production of reactive oxygen species or reduced antioxidant reserves is responsible for a majority of DNA fragmentation (almost 80%) seen as a result of infection, inflammation or in cases of various clinical diagnosis of male infertility [61-73]. DNA fragmentation as a result of single or double strand breaks can be measured by two common methods i.e. sperm chromatin structure assay (SCSA) [74,75], or by the terminal deoxynucleotidyl transferase-mediated dUTP nick end-labeling (TUNEL) assay [76]. TUNEL assay however cannot differentiate between apoptosis and necrosis.

Apoptosis in sperm is different from apoptosis seen in somatic cells where it is regulated at the plasma level (presence of Fas receptors), nucleus (presence of p53 inducing upregulation of Bax gene and down regulation of Bcl-2 expression) and cytoplasm (activation of Bax and release of cytochrome c and caspase cascade in the cytosol) [77-79]. Ejaculated sperm show features characteristic of apoptosis such as ultrastructural observation of the chromatin, mitochondria, the nuclear envelope, plasma membrane, presence of apoptotic bodies and presence of DNA fragmentation and externalization of phosphatidyl serine residues.

Abortive apoptosis like features in immature/abnormal sperm include remnants of cytoplasm and poor chromatin packaging and/or damaged DNA Abortive apoptosis is initiated during spermatogenesis. Spermatozoa earmarked for elimination escape at ejaculation in what is called abortive apoptosis and contribute to poor sperm quality. This is largely due to the presence of excess cytoplasm present in morphologically abnormal sperm [80-82]. More than 40% of the cells earmarked to be eliminated were reported to be present on the seminal ejaculate as examined by Annexin V and TUNEL assay [83].

DNA damage can also result from activated PARP and activated caspase3. PARP-1 has been implicated in DNA damage and apoptosis, in addition to its more complex events such as nucleosome binding property that promotes formation of compact, transcriptionally repressed chromatin structures. It is also linked with nuclear restructuring when nucleus is compacted with the introduction of protamines. It activates apoptosis during dramatically increased DNA repair and damage. Cleaved PARP also provides an early marker of detecting apoptosis as cleavage of PARP-1 occurs before DNA fragmentation [84].

DNA damage in ejaculated spermatozoa cannot be explained by apoptosis alone [80,82]. DNA damage can also be due to aneuploidy as well as mutations, chromosomal disjunction and meiotic segregation [85-87].

A study conducted by Moskovtsev et al. showed that as the incidence of semen abnormalities increased in infertile men, the extent of DNA damage also increased concomitantly [88]. Many other studies have reported a positive correlation between increasing male age and DNA damage [6,89]. Using DNA fragmentation Index (DFI) as an index to measure DNA damage/fragmentation, Moskovtsev et al. reported that in comparison to age group <30, age group which was ≥ 45 had twice the DFI (15.2% vs. 32.0%). DFI levels for 30–35, 35–40 and 40–45 were found out to be 19.4%, 20.1% and 26.4% respectively [6].

Similar results were shown in a study conducted by Singh et al. in which it was shown that the percentage of highly damaged DNA sperm in age group 36–57 years was significantly higher compared to the age group 20–35 years [89]. In another study involving 215 couples, it was shown that sperm DNA damage doubled from paternal age of 25 to 55 years [90]. A positive association was reported between DFI and increasing paternal age [89-91]. In a group of men with normozoospermia, the DFI level increased by 5% in age group ≥40 compared to age group ≤40. A similar trend was seen in a group of men with oligoasthenoteratozoospermia in which age group of ≥40 had 8% higher DFI levels compared to age group ≤ 40 years [89]. Barroso et al. proposed that the association between DNA fragmentation and advanced paternal age is present due to sperm chromatin defects [92].

A recent meta-analysis study comparing 26 studies involving 10,220 subjects, the authors reported a significant negative association of male age with DNA fragmentation [93]. They advocate the routine screening of men with advanced age for DNA fragmentation as well as cautioning patients of the potential risks. Ageing male and its effect on the functional capacity of the sperm as measured by phosphatidyl serine expression have been reported [94]. Significantly higher expression of phosphatidyl serine translocation at the sperm membrane indicative of apoptosis was reported in men 40 y and older. Similarly a trend was also reported in sperm DNA damage and increasing age of the male.

### DNA integrity and ART outcomes

Sperm DNA damage is associated within lower probability of conception and a longer time to conception [90,95,96]. These studies suggest that DNA damage is a better predictor of pregnancy than the conventional semen parameters [95]. Also DNA damage is correlated with lower pregnancy rates in intrauterine insemination and conventional IVF but not intracytoplasmic sperm injection (ICSI) lower pregnancy rates [60,97-101]. In a recent study by Nij’s and his group, a prospective study consisting of 278 patients who underwent intracytoplasmic sperm injection (ICSI) or *in vitro* fertilization (IVF) was examined for an association between semen parameters and men’s age. No significant influence of male age was reported on the fertilizing capacity [102].

Positive correlations have been reported between an increased sperm DNA fragmentation, reduced motility and ART outcomes leading to lower pregnancy rates and higher miscarriages [103]. Such DNA integrity reduction was shown to be correlated to advanced paternal age (especially for ages beyond 40 years) [6,89], supporting the overall negative effect of ageing fathers on IVF/ICSI success rate and hence ART outcomes [104,105].

Sperm DNA integrity is not only important for successful IVF but also for normal embryonic development. It has been recently shown that the advanced paternal age and its adverse effects on sperm DNA integrity also interfere with early embryonic development. Morris et al. showed that sperm DNA damage was strongly associated with men of age 29–44 years as well as with impairment of post-fertilization embryo cleavage [106]. In another study of 132 ICSI patients with father’s age of >40 years, sperm DNA fragmentation was significantly affected post-implantation during embryonic development [107]. In a cross-sectional study of 215 infertile men who underwent ART, Simon et al. showed that increased sperm DNA damage negatively affected early embryonic development and significantly reduced subsequent implantation [108]. In study comprising of 1023 infertile couples, Frattarelli et al. was able to show that sperm from men >50 years led to normal embryonic early cleavage but showed a decrease in blastocyst formation rate [109].

Two large studies have shown that paternal aging is associated with increased risk of pregnancy loss after an established pregnancy by IUI suggesting that advanced paternal age may affect genomic integrity and thereby negatively impact the embryo development [60,110]. A lack of consistent significant association between paternal age and sperm concentration as well as lack of association between paternal age and IVF or ICSI pregnancy rates [60,110-113].

Contrary to this another meta-analysis report consisting of 7 IVF and IVF/ICSI studies reported no association of paternal age with pregnancy loss after an established pregnancy [113]. This could possibly be due to the fact that the natural and IUI pregnancy in these studies were from men with relatively homogenous and normal semen parameters whereas those in IVF/ ICSI were from a heterogeneous population of men and this may have diluted the effect of age.

In conclusion, advanced paternal age increases the DNA fragmentation in sperm negatively affecting the IVF/ICSI success rates, ART outcomes as well as early embryo development. Despite increasing evidence of positive correlation between sperm DNA fragmentation and reduced male infertility, the ASRM guidelines does not support the routine use of sperm DNA integrity assessment in clinical practice [100]. However, they recommend further confirmation of sperm DNA integrity test using randomized studies and a high number of patients.

### Telomere length

Telomeres are tandemly repeated hexameric nucleotide repeat sequences (TTAGGG). Telomeres cap the ends of eukaryotic chromosomes. Their primary role is to preserve genomic structure and maintain its stability [114]. With each successive cell division, and hence with aging, the telomere length in somatic cells undergoes progressive shortening [115-119]. The somatic cells, for years were represented by leukocytes, but in a recent study conducted by Daniali et al. [120] four different somatic cells (leukocyte, muscle, skin and fat cells) were used to measure the association between telomere length and increasing age [120]. Like leukocytes, three other somatic cells’ telomere length was also found to decrease with increasing age [120]. In somatic cells, the guanine rich repetitive telomere DNA is maintained by telomerase, a reverse transcriptase enzyme [121]. With each cell division, some telomere repeats are not copied and hence are lost. But telomerase extends telomere by adding TTAGGG repeats. With increasing age, the incomplete DNA replication leads to telomere shortening [121]. When telomere length reaches a critical length, the cell cannot divide and the cell enters cell-cycle arrest or undergoes apoptosis. Telomere length is maintained by telomerase that is maximally expressed in highly proliferative cells such as germ cells and neoplastic cells [122,123]. A strong positive correlation has been reported between paternal age at birth and offspring LTL [124-129].

It has been reported that increased Leukocyte Telomere Length (LTL) is associated with reduced risk of atherosclerosis and hence, increased survival. Since increased paternal age increases LTL, it is a possibility that offspring of relatively old fathers have reduced risk of atherosclerosis and increased survival [130]. Consequently, increased LTL can also increase the risk of breast cancer in daughters of old fathers since it has been reported that there is a correlation between increased LTL and increased breast cancer risk [128].

Interestingly and compared to somatic cells, sperm (germ cell) telomere length was found to increase with increasing age [127,131,132]. Although such rare mechanism of telomeres’ extension remains unclear and poorly understood, it might be explained as kind of a biological resistance against the aging process. This molecular resistance expressed by human species against aging might be necessary to boost the chances of perpetuation of the species’. Further studies are required to confirm this discrepancy of telomerase extension observed in testis. In fact, it has been reported that average telomere length is heritable and can be passed down to offspring [126]. Interestingly, the effects of paternal age on telomere lengths have also been noticed in offspring [133]. It has been shown that telomere length inheritance is mainly determined by an offspring’s father [134]. A meta-analysis comprising 19,000 participants was conducted by Broer et al. [7]. These investigators analyzed six studies where they randomly examined telomere length and its heritability. A negative association was reported between telomere length and the age [7]. A significant correlation was also seen between advanced male age and telomere length, though maternal age played a more significant role [7].

In offspring both sperm [133] and leukocyte telomere length increases with increasing paternal age [129,130,133]. The role of sperm telomeres and telomere length is still unclear. Although both leukocyte telomere length (LTL) and sperm telomere length (STL) correlate within the same individual, LTL decreases whereas STL increases with age [127,131,132,135]. This is more likely related to the increased activity of reverse transcriptase activity - the catalytic unit of telomerase [136,137]. High reverse transcriptase activity in germ cells or cellular attrition resulting in death of stem cells with shortened telomere length results in selection of sperm with longer telomeres [127,133].

The role of STL in spermatogenesis or fertility potential is unclear. A recent report examined a group of healthy 18–19 years old subjects and compared telomere length and sperm, spermatogenic activity and the age of the parents at birth [132]. They showed a positive correlation between STL and sperm count and significantly shorter STL in men with oligozoospermia when compared to those with normozoospermia. They also showed effect of parental age on offspring STL [132]. In another study, STL in men with idiopathic infertility and controls was examined and a shorter telomere length was reported in men with unexplained male infertility [138]. Although there were differences in these two studies mainly, in the study by Thilagavathi et al., LTL was not considered and included low number of subjects with unknown age and normal mean sperm count, sample size was small compared to the study by Ferlin et al. It is clear that telomeres play an important role in meiosis and thereby maintain genomic integrity [139]; shorter telomere suggested impaired spermatogenesis through segregation errors as telomerase activity peaks in the testis in meiosis I primary spermatocytes [139]. Shorter telomeres can be regarded as putative cause of impaired spermatogenesis and male infertility, although additional studies are needed to verify this interpretation. Shorter telomeres in ejaculated sperm may be a marker of damaged spermatogenesis and a consequence rather than a cause of altered spermatogenesis. Shorter telomere length in oligozoospermic men as reported by Thilagavathi et al. has implications in assisted reproductive techniques as the offspring will inherit smaller [138]. However additional studies are need to verify the pathophysiological link between STL and damaged spermatogenesis as well as its effect on the offspring telomere length especially in older couples where the man is oligozoospermic.

### DNA mutations

In contrast to oogenesis, sperms divide (or spermatogenesis occurs) continuously throughout reproductive lifetime and hence accumulates greater number of cell divisions. Spermatozoa a can also acquire de novo single nucleotide variants or mutations because of the continuous ongoing process of spermatogenesis that involves multiple asymmetric pre-meiotic spermatogonial divisions and the testicular environment is more prone to toxic effects of oxidative stress in ageing men [8]. Furthermore errors on post-meiotic remodeling of chromatin remodeling and DNA repair cam also result in de novo mutations [140]. Spermatozoa from aging fathers can also be more prone to chromosomal aneuploidy [141]. The paternal contribution to offspring novo mutations was estimated to increase by 4% per year [142]. At the age of 20, a sperm would have undergone 150 chromosomal replications, and at the age of 50, it would have gone through 840 replications [8,143,144]. This increases the probability of replication errors in the germ line leading to the accumulation of mutations and hence increased *de novo* mutation rate in spermatozoa [142]. This problem is further aggravated when age-sensitive processes such as DNA replication and repair are compromised due to an increasing age [8]. Kong et al. and his team reported the positive association between the age and *de novo* mutation rate [142]. On average, the rate of *de novo* mutation increases by two base pairs every successive year [142]. Kong et al. also reported that the heritability of mutations in an offspring is mainly attributed to paternal age [142]. This increases the probability of older fathers conceiving fetuses with rare and harmful disorders [145]. Paternal Age Effect (PAE) disorders are a small number of rare disorders which occurs due to specific mutations in fibroblastic growth factor receptor (FGFR) [146-148]. Wyrobek et al. found that sperm of men with age of 22–80 years associated with mutation in FGFR3 in particular and this was also associated with achondroplasia [149]. An increasing paternal age is one of the major sources of mutations found in human species [8]. Though this phenomenon aids in the diversification of the species, unfortunately, it can also increase the incidence of rare disorders in the human population.

A chromosomal anomaly such as Klinefelter syndrome, 47, XXY is carried by 5% of all infertile men and microdeletions of the long arm of the Y chromosome are present in 10% of azoospermic or severely oligozoospermic men [150]. It has been shown that the post-meiotic events during spermiogenesis are critical from which *de novo* genetic mutation could be induced [140]. A number of mechanisms have been suggested to explain the induction of these *de novo* mutations. Among these is a base substitution due to the nucleotides are not incorporated by the polymerase [151], and insertion or deletion which could lead to a high rate of cell divisions and subsequent *de novo* mutation [152]. It is interesting to mention that the frequency and the increase in a *de novo* chromatin translocation detected in 10 sperm donors was found to be not an age dependant [153] suggesting a replicate-independent mechanism for formation of the translocations. NRA51 nuclear receptor also called the steroidogenic factor 1 is a key transcriptional regulator of genes. Mutations of NRE5-1 have been reported in 46,XY disorders of sex development and in 46,XX primary ovarian insufficiency in 4% of men with otherwise unexplained severe spermatogenic failure [154]. Some forms of male factor may be an indicator of testicular dysgenesis which requires careful clinical investigation of men presenting with infertility and inconsistent testosterone and gonadotropin levels. De novo point mutation in the Y-chromosomal gene *USP9Y* has been reported in a man with non-obstructive azoospermia, causing spermatogenic failure [155]. Similarly these authors also reported a single-gene deletion that was associated with spermatogenic failure.

### Chromosomal aneuploidies

Chromosomal aneuploidy is the presence of an abnormal number of chromosomes in a cell. Chromosomal aneuploidy is caused in a sperm when it undergoes meiosis but the chromosomes are not equally divided in daughter cells because of disjunction. Most of the aneuploid embryos die *in-utero* and hence chromosomal aneuploidy is the leading cause of failed pregnancy [156]. However, 1% of aneuploid pregnancies lead to live birth [156] which accounts for a large number of congenital birth defects and/ or mental retardation [157].

On average, 10% of sperm cells of healthy male population have chromosomal aneuploidies and include chromosome 21 and 22 [158]. However, this number increases with paternal age [159]. The incidence of sex chromosome disomy 18 significantly increases among older men (>50 years) when compared to younger men [159]. McIntosh et al. reported increased risk of up to two fold among fathers of 50 years and older when compared to the fathers of age group 25–29 years [160]. Table 3 summarizes the effect of paternal age on chromosomal aneuploidies.

**Table 3:** **Effects of paternal age on some of the chromosomal aneuploidies**

**Type of chromosomal aneuploidy**	**Relative risk**	**Reference**
Trisomy 21	↑	[160]
Trisomy 18	Mixed	[161-163]
Trisomy 13	Mixed	[161]
Trisomy 16	No affect	[164]
Trisomy 15	No affect	[165,166]
47,XXY	Mixed	[167]
45, X	Mixed	[168]

### Molecular aging and genomic instability

**Figure 1:**
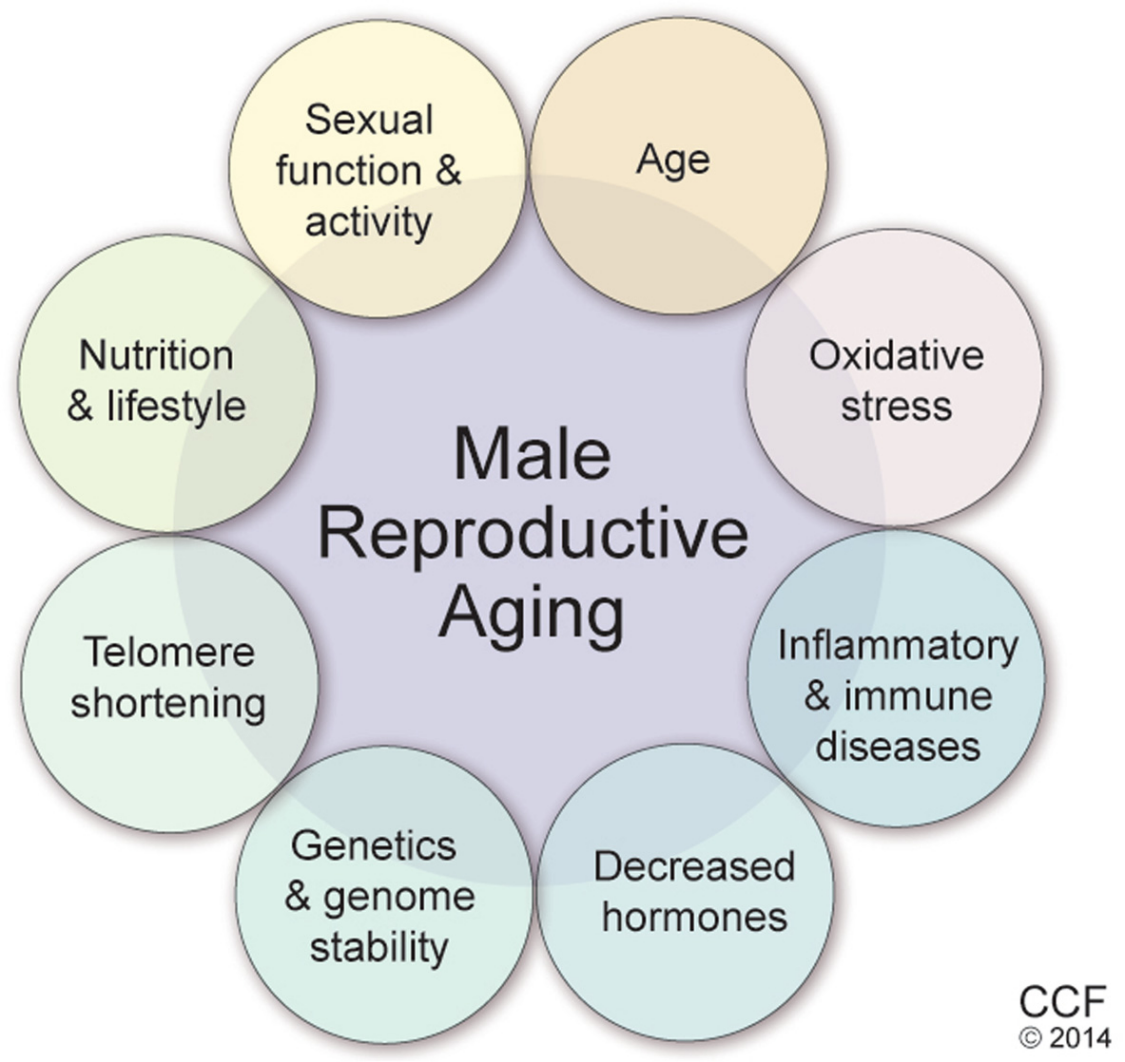
Main factors involved in impaired male infertility due to reproductive aging.

Aging is a multifactorial and complex process leading to progressive impaired cellular functions and hence increased vulnerability to diseases [169]. Aging affects several processes including DNA damage [170], telomere shortening [171,172] leading to cellular senescence or apoptosis [172] (Figure 1). In this context, advanced paternal age would lead to the accumulation of *de novo* mutations, male infertility and increased genetic risks on the offspring e.g. autism and schizophrenia [142,173]. The dysfunctional telomerase was reported to induce DNA-damage response in senescence phase [174].

Genomic instability at the cellular level will lead to variation at the gene expression level and affect microRNA (miRNA) patterns with aging [172,175,176]. miRNAs are non-coding RNAs consisting of small RNAs (~22 nucleotides) and are critical regulators of post-transcriptional gene expression by targeting mRNAs for cleavage or translational repression. These miRNAs have been identified in the seminal plasma as potential markers of male infertility and their expression patterns change with age or other stress factors as vasectomy [177,178]. Therefore, more work is needed at this level to enhance our comprehension of the gene players controlling normal versus abnormal sperm development, differentiation and maturation in both adult and aged cases. The final differentiation and maturation of spermatozoa occur in the epididymis where the coiled mass of tubes play crucial role in carrying, storage and maturation of sperm [179,180]. Zhang et al. carried out comparative expression pattern analysis of microRNAs in epididymis of newborn, adults (aged 25 years) and aged (aged 75 years). The analysis revealed that a total number of 251 miRNAs expressed in newborn epididymis (represents 63% of the known miRNAs) was dropped to 31% in the aged case [181]. The mechanism through which this change in miRNAs expression affects the sperm quality and DNA integrity is to be yet investigated.

### Epigenetics of male aging

Epigenetics is stable heritable modification on histone tails but not the DNA sequence that leads to altered gene expression [182]. Unlike DNA mutations, epigenetic patterns can be disrupted or silenced by various environmental and endogenous factors such as nutrition, age, drug/toxin exposure and phenotypic variation. Therefore, both spermatogenesis and spermiogenesis processes are marked by successive steps of epigenetic reprogramming of the male gamete which is influenced by several environmental factors (Figure 1). These epigenetic events may impair or inhibit key steps of fertilization, implantation and/or the embryo development [183]. Loss of methylation at the paternally imprinted H19-DMR (differentially methylated region) locus was reported in sperm of men with unexplained low sperm counts [184]. On further investigation revealed abnormalities in 14% to 20% of men with moderate or severe oligozoospermia [185-188]. Genome wide analysis suggested global hyper methylation of DNA from poor quality sperm, pointing to the poor improper erasure of DNA methylation during germ cell development [189]. Epigenetic modifications in the sperm selected for ART can also lead to perturbations or increase the imprinted congenital phenotype because of the ART technique itself. Methylation profile of two imprinted loci H19-DMR and PEG 1/MEST-DMR have been studied in men showing phenotypes ranging from severe oligozoospermia to normospermia. The methylation profile of these two loci was used as a marker of sperm DNA methylation status by Montjean et al. [190]. They found epimethylation and epimutations in 20% in H19-DMR and 3% in PEG 1/MEST-DMR of spermatozoa of oligozoospermic men but did not observe an association with the genetic variants or in the ART outcome.

It has been reported that in addition to the age, the role of father’s nutrition and his exposure to toxicants is so strong that not only affects his offspring’s epigenetic factors but also his grand-offspring epigenetics factors as well [10]. However, a study conducted by Benchaib et al. reported that there is no correlation between DNA methylation and paternal age [191,192]. This study proves that some of the epigenetic factors are not only heritable but also stable.

DNA methylation and repressive histone modification are two of the most common mechanisms which cause gene silencing. It has been found that DNA methylation plays an important role in mammalian development and influences different processes like X-inactivation [193], genomic imprinting and embryo development as soon as the zygote is formed [194].

To further prove the importance of DNA methylation in embryo development, Benchaib et al. [192] conducted a prospective study to assess the influence of global sperm DNA methylation on IVF outcomes. They demonstrated that pregnancy outcomes were significantly improved in sperm with global methylation level (GML) higher than arbitrary threshold value (555 AU). However, others reported no change in fertilization rates and quality of embryos [191,192]. These investigators suggested that germ line which has been epigenetically reprogrammed might lead to compromised spermatogenesis and eventually result in infertility. Ace-1(Ace-variant1), Prm1 (Protamine 1), Prm2 (Protamine 2) and Smcp (Sperm mitochondrial-associated cysteine-rich protein) are key sperm genes which are known to bind to chromatin. A recent longitudinal study conducted on mice reported that expression levels of Ace-1, Prm1, Prm2 and Smcp genes which are genetically regulated by epigenetic factors were shown to decrease with increasing paternal age. During spermiogenesis, these proteins replace most of the canonical histones [194]. Decreased expression levels of Prm1 Prm2, Smcp result in decreased semen quality and IVF pregnancy rates [195].

Furthermore, the levels of 5-mc and 5-hmc (methylated forms of cytosine) increased (by 1.76% every year) with a concomitant increase in paternal age in donors which in turn causes gene silencing [196]. Angelman Syndrome is a neurogenetic disorder associated with both developmental and intellectual disability [197] while Bechwith-Wiedmann Syndome is a genetic disorder which is usually associated with overgrowth and increased risk of childhood cancer [198]. Gosden et al. reported that incidence of rare disorders like Angelman syndrome and Beckwith-Wiedemann syndrome increased significantly in babies conceived with different assistive reproductive techniques, suggesting that the result is possibly because relatively old couples opt for assisted reproductive technology (ART) techniques for conception [199].

### Paternal Age Effect (PAE) disorders

The correlation between increasing paternal age and genetic defects was first suggested in late 1800s by Weinburg [200] while the association between increasing paternal age and genetic disorder such as Achondroplasia was found by Penrose in 1955 [201]. Ever since, many other genetic disorders have been associated to increasing paternal age. An increase in *de novo* mutation rate has been reported as the major cause of paternal age effect disorders (Figure 1). Most of the mutations detected in disorders associated with increasing paternal age are single base pair substitutions [202]. In a study conducted by Kong et al. using deep sequencing analysis, they reported that with increasing paternal age, the germ line single base pair substitutions increased at the rate of 2 base pairs per year [142]. Realizing the significance of paternal age disorders in male, the British Andrology Society and American Society for Reproductive Medicine set the upper age limit for sperm donors at 40 years [203,204].

In this section we will highlight some of the genetic disorders which are associated with advancing paternal age.

### Schizophrenia

Advanced paternal age has been associated with schizophrenia in many studies [14,205-208]. Schizophrenia is a psychiatric disorder which is associated with disabilities in social and occupational functioning. It also involves recurrent or chronic psychosis [209]. Schizophrenia is not only lethal in terms of the disability caused to a victim, but also an economically burdening disorder. It has been ranked by WHO as one of the top ten diseases contributing to global burden of diseases [210]. Schizophrenia is an etiologically heterogeneous syndrome and has a strong genetic influence [211,212]. The genetic influence is so strong that a quarter of all cases of schizophrenia are attributed to increasing paternal age [213].

In a cohort study comprising of 754,330 Swedish subjects, it was reported that with every 10 year increase in paternal age at the time of conception, the risk of an offspring having schizophrenia increased by 1.47 times. Interestingly, offspring with younger fathers (<21 years) were also at a higher risk of schizophrenia compared to the fathers aged 21-24years at the time of conception [206]. In a meta-analysis conducted by Miller et al. comprising of 12 cohort and case–control studies, the offsprings of older fathers (>30 years) had higher risks of schizophrenia compared to reference paternal age of 25–29 years. Similar to the result of Swedish cohort study mentioned earlier, Miller et al. also showed that younger fathers (<25 years old) had higher risk (Relative Risk ratio = 1.08, 95% CI: 1.02–1.14, P = 0.01) compared to the age group of 25–29 years old fathers [207].

To further investigate whether socially and culturally different countries showed similar association between advanced paternal age and schizophrenia, Tsuchiya et al. and his team conducted a study in Japan and reported a similar association [214]. Wohl et al. conducted a study to compare the effect of different paternal age groups on risk factor of schizophrenia in offsprings. The odds ratio for fathers’ age increased exponentially from 1.16 in age group 25–34 years to 5.92 in fathers over the age of 55 years [215].

Frans et al. conducted a national register-based cohort study which involved 120,758 individuals to examine whether grandparent’s age contributed to the grand offspring’s risk of having schizophrenia. It was observed that old grandmother’s age increased the risk of schizophrenia in grandchild but not grandfather’s age [208].

It has been reported that the association between paternal age and schizophrenia is mainly due to the accumulation of *de novo* mutations in sperm [206,208,211,212]. Although a number of studies have supported the mechanism of *de novo* mutations as a causative factor for the occurrence of Schizophrenia, other mechanisms may play a role. For example, when the age of a father was adjusted for first fatherhood, no association was found between increased paternal age and increased risk [216].

Dysregulation of epigenetics at the DNA methylation, histone modifications or chromatin remodelling level, with respect to increasing paternal age could also increase the risk of Schizophrenia. Genomic imprinting also known as parental imprinting is a phenomenon in which a gene is expressed in a parent of origin-specific manner [217]. Alterations in epigenetic mechanisms like parental imprinting can also have negative implications on the offspring [218].

### Bipolar disorder

Bipolar Disorder (BPD) is a heterogeneous brain disorder associated with severe mood swings. Many studies have shown significant association between risk factors of BPD in offspring’s with increased paternal age [14,219,220].

A population based registry study involving 7, 328, and 100 individuals conducted by Frans et al. found out that the risk for BPD in offsprings increased with increased paternal age [219]. In comparison to offsprings of fathers aged 20–24 (control group), the offsprings of fathers aged 55 and older had 1.34 times higher risk of being diagnosed with BPD [219]. Offspring’s whose fathers were <20 years old at the time of their birth had 2.63 times higher risk of being diagnosed with BPD [219]. The reasons behind increased risk of BPD in younger population can be due to immature sperm, stressful environment, smoking and even alcohol abuse. Also, younger fathers are likely to come from disadvantaged background, which can contribute to poor postnatal care [209]. Menezes et al. showed that with increase of every ten years in paternal age, the risk factor for BPD in offspring increased by 1.20 times after adjusting for maternal age [220].

In a recent population-based cohort study which involved 2,615,081 individuals from Sweden, it was reported that increased paternal age was associated with increased risk of bipolar disorder. The offsprings born to parents >45 years old had increased hazard ratio (or relative risk ratio) of 24.7 compared to off springs born to parents 20–24 years old [14]. In contrast, Buizer-Voskamp et al. did not find any association between advanced paternal age and increased risk factor of BPD [205].

Similar to schizophrenia and other mental disorders associated with increasing paternal age, BPD might possibly result from *de novo* mutations which are caused by DNA copy errors. Epigenetics might also play role in causing paternal age effect disorders, [209,221]. Kaminsky et al. [222] reported that compared to the control group, the DNA methylation of human leukocyte antigen [223] complex group 9 gene (HCG9) increased in BPD patients. This might explain the possible mechanism of the occurrence of increased risk factor for BPD for offsprings with advanced paternal age since, DNA methylation increases with advanced paternal age.

### Autism

Autism spectrum disorder refers to a group of complex disorders which are characterized by difficulties in verbal and nonverbal communications, interaction with people and tendency to display repetitive behaviours [224]. Autism is usually diagnosed in children at an early age of 3 years [225].

Many studies have shown that there is a significant association between increased paternal age and the risk of autism [14,205,226-229]. In a recent registry study conducted by Buizer-Voskamp and his group, it was calculated that in comparison to younger fathers (<20y), older fathers (>45y) had 3.3 times higher risk of conceiving an offspring with autism [205]. Similarly, Reichenberg et al. [226] reported that compared to offspring’s of parents who were <30 years old, the offspring’s of parents >50 years had 5.75 higher risk of having autism. Another study performed on Icelandic population concluded a statistically significant correlation between increasing paternal age and autism [142]. In a meta-analysis conducted by Hultman et al. it was found that the risk of autism in offspring increased with advanced paternal age [227]. Compared to reference age group (<29y), the risk of autism increased two fold in offsprings of age group >50 years, while controlling maternal age and other risk factors [227].

Interestingly, a significant association was also found between advanced grandpaternal age at the time a parent was born and the risk of autism in grandchildren. An offspring would have 1.79 times increased chance of having autism if his/her maternal grandfather gave birth to his/her mother when he was over the age of 50 years. For an offspring with paternal grandfather, the risk is reduced to 1.67 times but it is still significantly higher [182,230]. Using an animal model, a similar association was found in mouse which displayed decreased sociability, increased grooming activity, increased ultrasound vocalization (USV) activity and increased anxiety-like responses in offsprings of grandfather who gave birth to their parents at an older age [231].

It is believed that one of the causative factors of autism is mutation of transcription factors which play dominant role in gene expression [224]. As discussed earlier, epigenetic factors have a very high heritability and this might explain the reason why higher risk of autism is found across the generations. Some studies have also proposed that age-related *de-novo* mutations in male germ contribute to increased risk of neurodevelopmental disorders like autism [232,233].

### Other disorders

We have discussed the effect of paternal age on different neurocognitive disorders. Other specific conditions ranging from autosomal disorders such as Achondroplasia and Apert Syndrome to various congenital anomalies like Klinefelter syndrome have been associated with increasing paternal age. Some of the most common disorders associated with advanced paternal age are shown in Table 4.

**Table 4:** **Effect of paternal age on various disorders showing effect of age and relative risk ratio**

**Type of disorder**	**Disorder**	**Age (Reference age)**	**Relative risk**	**Reference**
Neuro-cognitive	Autism	>45(<20)	3.3	[205]
		>50(<30)	5.75	[226]
		>50(<29)	2.2	[142,227]
	Bipolar disorder	>55(20–24)	1.34	[219]
		Not specified	1.20	[220]
		>45(20–24)	24.7 (Hazard Ratio)	[14,206]
	Schizophrenia	Not specified	1.47	[207]
Autosomal dominant		>50(25–29)	1.66	[214,215]
		>32(<28)	3.00	[13]
		>55(25)	5.92	[211,213,216]
	Achondroplasia	>30(<30)	3.48	[234]
		>50(25–29)	7.80	[236]
	Apert syndrome	-	-	[235-237]
	Neurofibromatosis I	>35 (<35)	1.69	[238]
		>40(<30)	2.9	[239]
	Osteogenesis imperfecta	22-80	1.37	[13]
		>35(<35)	1.62	[240]
		>35(<25)	-	-
	Retinoblastoma	>35(Not specified)	1.73	[241]
		>45	3.00	[242]
Congenital Abnormalities	Cleft Lips	Not specified		[243]
	Anencephaly	>40		[11]
	Transposition of Great Vessels	>45 > 40	1.27	[11]
			1.20	
	Ventricular Septal Defects	>35	3.63	[160]
		30–34	1.69	
		(25–29)		
	Artrial Septal Defect	35-39	1.95	[160]
		(25–29)	1.2	
		40–44		
		(25–29)		
	Neural tube defect	45-49	1.3	[160]
		(25–29)		
		>50(25–29)	1.6	[160]
		35-39	0.6	[244-246]
		(20–29)		
		>50(25-29	2.3	[244-246]
	MSA	>35(30–34)	1.33	[246]
	Tracheoesophageal fistula	30-34(<25)	2.55	[247]
		-	3.12	[248]
		-	1.34	[248]
Others	OCD	>40	1.14	[248]
	Childhood CNS Tumor	>35-39	1.11	[248]
		(25–29)		
	Childhood Leukemia	>35-39	1.29	[248]
		(25–29)		
	Mood disorder	>35-39	1.07	[249]
		(25–29)		
	Personality disorder	--	-	[249]
	Mental retardation	-	-	[249]
	Pervasive developmental disorders	-	-	[249]

### Reproductive and fertility outcomes

#### Advanced paternal age and time to pregnancy/male fecundity

Fecundity is defined as the likelihood of achieving a pregnancy in a defined period of time. Using time to conception as an index to measure male fecundity, Ford et al. [250] reported that there is a significant decline in male fecundity with advanced paternal age after adjusting maternal age and other confounding factors. For men older than 40 years, the odds ratios for conception in <12 months were 0.62 for 30–34 years old, 0.50 for 35–39 years old and 0.51 compared to the reference age group (<25 y) [250]. However, one of the limitations of this study was that it was unable to determine whether the male fecundity reduced solely because of the biological changes in male reproductive system or because of reduced coital frequency, which is also associated with increasing paternal age [21-24]. Association of paternal age with fertility is contradictory. This may be attributed to the decline in male sexual activity as frequency of intercourse decreases with age. The general consensus is that paternal age is associated with reduced fertility especially in couples where men are older than 40 years and age of the women is at least 35 years [251,252]. To find the effect of biological changes in failure to conceive, de La Rochebrochard et al. [204] conducted a study to find the association between advanced paternal age and the risk of failure to conceive after IVF attempts. They reported that the odds ratio of 1.70 for paternal age of 40 years and older compared to 30 years and younger, showing significant increase in failure to conceive [204]. When maternal age was involved, the odds ratio increased to 2.00 for men >40 years when the woman was 35–37 years and the odds ratio increased exponentially for same paternal age when the maternal age increased to >41 years [204].

In a similar study conducted by Hassan et al. it was reported that compared to men who were <25 years, older men who were >45 years had 4.5 times and 12.5 times increasing risk of having Time to Pregnancy (TTP) of >1 years and >2 years respectively [253]. Dunson et al. also reported significantly reduced fertility in men >35 years [254].

### Paternal age, intrauterine insemination success and live birth rates

Reports have shown a decrease in assisted pregnancy rate with increasing paternal age, [255-257]. Mathieu et al. reported that male age ≥ 35y was associated with decreased clinical pregnancy rate [255]. Belloc et al. reported that significant decline in artificial conception rate when pregnancy rate decreased from 12.3% per cycle in men aged <30 to 9.3% in men ≥45 years Belloc et al. [256]. Similar findings were reported by Demir et al. [257]. In a prospective study conducted by Klonoff-Cohen et al. it was reported that with increasing paternal age, the live birth rate decreased, showing a decrease in artificial pregnancy rate [258].

### Spontaneous abortions

Spontaneous abortion is defined as loss of pregnancy occurring before 20 weeks of gestation [158]. It is seen in 10-15% of clinically recognized pregnancies [259]. Increasing paternal age is significantly associated with the risk of spontaneous abortions [260]. In a retrospective study, de la Rochebrochard and her colleagues [260] reported that compared with 20–29 years age group (both paternal and maternal), the odds ratio of risk of having miscarriage increased to 1.06, 1.31 and 1.80 when the paternal age increased from 30–34 years, 35–39 years and 40–64 respectively while the maternal age remained unchanged at 20–29 years. Similarly in another study, the odds ratio increased by 1.2, 1.5 and 1.3 for age groups 30-34y, 35-39y and >40 years old respectively, after adjusting for maternal age [261]. Slama et al. reported that subjects in >35 years group had increased risk of 1.27 times compared to <35 years age group [262]. In a recent French study, Belloc et al. reported an increase in miscarriage rate to 32.4% in fathers ≥45 years compared to 13.7% in the fathers who were <30 years [256]. However, not all studies showed similar results [113,258,263,264].

### Pre-eclampsia and advanced paternal age

Pre-eclampsia refers to the onset of hypertension and either proteinuria or end-organ dysfunction after 20 weeks of gestation in a previously normotensive woman [265]. Harlap et al. reported a significant association between increasing paternal age and preeclampsia [266]. These authors observed an increase in the odds ratio in 35-39y, 45-49y and 50-54y paternal age groups by 1.30, 1.89 and 1.54 respectively, compared to the 25-29y paternal age group and this was independent of maternal age [266,267].

### Pre-term birth and low birth weight and increasing paternal age

Pre-term delivery is defined by the occurrence of delivery before the completion of 37 weeks of gestation [268]. Pre-term birth is responsible for causing 27% neonatal deaths worldwide, leading to over a million deaths annually [269]. It is also associated with more than 70% of early life morbidity and mortality, making it one of the largest health problems in reproductive health [270]. Zhu et al. reported that with increasing paternal age, the risk of preterm births increased [271]. Compared with the reference age group 20–24 years, paternal age groups 25-29y, 35-39y, 40-44y, 45-49y and >50y showed increased odds ratio of 1.3, 1.4, 1.7, 1.6 and 2.1 respectively for pre-term birth [271]. Astolfi et al. reported that the odds ratio for preterm increased with increasing paternal age. They reported that father’s age group had increased odds ratio of 1.91 and 1.71 when adjusted for maternal age of 20–24 and 25–29 respectively [272]. However, some of the previously conducted studies did not find any significant association between paternal age and increased risk of pre-term, [273-276].

Low birth weight is a leading cause of infant mortality in the United States. It is associated with attention deficit hyperactivity disorder (ADHD), blindness, epilepsy, chronic lung disease, cerebral palsy, all of them leading to long term health problems [277]. Alio et al. reported that in comparison to the paternal age group of 25–29 years, age group >45 years had 19% increased likelihood of low birth weight and 13% increased risk of preterm (between 33 and 37 weeks of gestation) birth [12]. In another study, Reichman et al. conducted a cohort study in which they concluded that fathers aged ≥35y had 1.9 times increased risk of conceiving low-birth weight offspring compared to 20-34y group [9].

### Still-birth/ fetal death and increasing paternal age

Still-birth defines a fetal death that occurs prior to the expulsion from its mother [278]. Alio et al. conducted a study where the paternal age group >45 years had 48% increased risk of still-birth (utero-fetal death ≥28 weeks) compared to the 25–29 years group [12]. In a cohort study conducted by Nybo et al. it was found out that the pregnancies fathered by men aged 45–49 y had an increased risk of late fetal death (>20 weeks of gestation) with an odds ratio of 1.40 when adjusted for maternal age [279].

For pregnancies fathered by men aged ≥ 50 years, both the risks for early fetal death (≤20 weeks of gestation) and late fetal death increased with the hazard ratio of 1.38 and 3.94 respectively [279]. Alio et al. reported that in comparison to the paternal age group of 25–29 years, age group >45 years had 22% increased risk of stillbirth [12]. Similarly, Astolfi et al. reported that father’s aged ≥ 40y contributed to increase stillbirth risk compared to fathers in younger age groups [280].

### Genome-wide association studies and male reproductive aging

The post-genomic area is marked by the development of cutting edge technologies that allowed a wide screening of the whole human genome at once. These genome-wide association studies (GWAS) have been widely used to study complex traits and to identify key genomic regions associated to several diseases. In this context, more than 1000 male infertility-associated genes have been already reported [281]. However, the transcriptomic, genomic and epigenomic behavior of these genes as well as many single-nucleotide polymorphisms (SNP) during the male reproductive aging is still unknown. A gene discovery approach based on hybridization/ microarrays technologies and followed by specific target identification using high throughput sequencing are required to further our comprehension of the molecular mechanism and signaling pathways underlying the male reproductive function in general and specifically the aging process [282]. A suitable choice of the type of tissues/fluids, the stage, and the factors to be investigated are also key elements to be considered (Figure 1), [283-285].

The GWAS technique has the potential to unravel many genetic disorders through the analysis (sequencing) of the DNA, RNA, miRNA, SNPs, copy number variations (CNVs), insertions/deletions and other genomic parameters related to male infertility and aging, [105,145,286]. However, such studies require a proper experimental design and enough number of patients with comparable characteristics which is challenging given the scarcity of the samples and the various aging effects to be assessed [169]. These suggested data are required to set up functional validation [281] to demystify the role of each target genes and understand the molecular process of male infertility in its entirety, at a particular stage and over time.

## Conclusions and future directions

Several studies have demonstrated the effects of increasing paternal age on various molecular mechanisms such as DNA mutations, chromosomal aberrations and epigenetic patterns. This molecular aging process was shown to induce changes in reproductive hormones’ profiles, decrease sperm quality parameters and contribute to male infertility. These alterations are also responsible for various types of congenital disorders and pregnancy outcomes such as spontaneous abortions and preterm births. Although a number of studies have been conducted to assess the negative effects involved with increasing paternal age, the molecular mechanisms which cause the effects are still poorly understood. It is proposed that further research should be conducted to demystify the mechanisms involved. The use of cutting-edge technologies mainly next-generation sequencing to study the relationship between aging and male infertility will build a framework for future studies on the molecular reproductive aging in order to design advanced male infertility diagnostic and therapeutic tools to delay the aging aforementioned negative effect. The identification of aging versus longevity-related genes will also help to predict the age impact on the reproductive function. Furthermore, it will be possible to accurately establish an ‘Age Threshold’which once crossed; a prospective father should attend a counselling session in which he should be educated about the risks involved with conceiving an offspring at old age.

## Abbreviations

Ace-1: Ace-variant1
ADHD: Attention deficit hyperactivity disorder
ART: Assisted reproductive technology
AU: Arbitrary units
BPD: Bipolar disorder
DFI: DNA fragmentation index
DHEA: Dehydroepiandrosterone
DHT: Dihydrotestosterone
FGFR: Fibroblastic growth factor receptor
FSH: Follicle-stimulating hormone
GML: Global methylation level
GnRH: Gonadotropin-releasing hormone
HPT: Hypothalamic-pituitary-testicular
ICSI: Intracytoplasmic sperm injection
IUI: Intrauterine insemination
IVF: *In vitro* fertilization
LH: Luteinizing hormone
LTL: Leukocyte telomere length
PAE: Paternal age effect
Prm1: Protamine 1
Prm2: Protamine 2
SHBG: Sex hormone-binding globulin
Smcp: Sperm mitochondrial-associated cysteine-rich protein
TTP: Time to pregnancy
USV: Ultrasound vocalization
WHO: World Health Organization
